# A meta-analysis of effectiveness of real-world studies of antipsychotics in schizophrenia: Are the results consistent with the findings of randomized controlled trials?

**DOI:** 10.1038/s41398-021-01636-9

**Published:** 2021-10-06

**Authors:** Lajos Katona, István Bitter, Pál Czobor

**Affiliations:** 1 Independent Researcher, Budapest, Hungary; 2 Department of Psychiatry and Psychotherapy, Semmelweis University, Budapest, Hungary

**Keywords:** Schizophrenia, Scientific community

## Abstract

Randomized controlled trials (RCTs) have been considered as gold standard for establishing the efficacy and safety of investigational new drugs; nonetheless, the generalizability of their findings has been questioned. To address this issue, an increasing number of naturalistic studies and real-world database analyses have been conducted. The question of how much information from these two approaches is congruent or discrepant with each other is of great importance for the clinical practice. To answer this question, we focused on data from the antipsychotic (AP) treatment of schizophrenia. Our aim was two-fold: to conduct a meta-analysis of real-world studies (RWS), and to compare the results of RWS meta-analysis with previously published meta-analyses of RCTs. The principal measure of effectiveness was all-cause treatment discontinuation for both RWS and RCTs (when not available, then drop out for RCTs). We included publications for 8 selected APs (oral formulations of amisulpride, aripiprazole, clozapine, haloperidol, olanzapine, quetiapine, risperidone, and long-acting injectable (LAI) risperidone). We identified 11 RWS and 7 RCT meta-analyses for inclusion. Our results indicated that the RWS yielded statistically conclusive and consistent findings across individual investigations. For the overwhelming majority of the comparisons where both RWS and RCT meta-analyses were available, there was good congruency between the RWS and the RCT results. Our results support that RCTs, despite their limitations, provide evidence which is generalizable to real-world settings. This is an important finding for both regulators and clinicians. RWS can provide guidance for situations where no evidence is available from double-blind clinical trials.

## Introduction

Antipsychotic drugs (AP) are recommended both for the short-term treatment of acute episodes as well as for the long-term maintenance treatment in schizophrenia [1]. The development of antipsychotics is regulated by competent authorities, which require evidence about efficacy and safety from randomized controlled trials for their approval [2–4]. As a consequence, RCTs, used during drug development, mostly do not yield direct head-to-head comparisons with the relevant treatments already available in clinical practice. The use and the exclusive use of placebo controls in schizophrenia studies has been challenged [3, 5].

Even though RCTs are considered to provide the highest grade of evidence, the question of generalizability of their results to real life outcomes arises for several reasons. These are, for example, the inclusion of highly selected non-representative samples in RCTs [6], fix doses, short duration, small sample size, and predominantly placebo control associated with regulatory recommendations [7]. While shorter duration in acute trials has been supported by data, which guides clinical practice [3], the number of long-term follow-up RCT studies is still small [8, 9]. Moreover, there is still a lack of clinically important real-world endpoints, such as hospitalization in these studies [10].

Most meta-analyses based on data from the registration studies as well as on post-marketing data do not overcome the problem of the lack of data for individual head-to-head comparisons since their outcome is an effect size against placebo, and less frequently against a standard comparator [11]. Network meta-analyses could potentially overcome this problem, i.e., they may yield data for comparative efficacy among medications, but they suffer from certain assumptions, including the generalizability of the efficacy estimates across all pair-wise comparisons, and the use of sparse data from the various nodes of individual comparisons [12]. An additional issue both for meta-analyses and network meta-analyses is the increasing placebo response rate in schizophrenia trials [13, 14].

To address the problem of generalizability of data of RCTs to real-world environments, two major approaches have been used: (1) naturalistic observational studies in real-world settings, and (2) large scale database analyses from healthcare databases.

Open label effectiveness trials are intended to address the questions as to how certain medications work in real-world clinical and non-clinical settings, but once again for reasons of feasibility these trials rarely include a wide range of medications. While they tend to focus on clinically relevant real-world endpoints, they are, nonetheless, somewhat limited by certain factors that also restrict RCTs (e.g., small sample size, lack of long-term follow-up) [15].

Database analyses of representative samples or of full, nationwide populations may hold-out the promise to overcome the above problems. These analyses rely on large samples, allow for head-to-head comparisons for a wide range of medications, can capitalize on endpoints with clinical as well as public health relevance, and make possible the investigation of long-term outcomes. Following patients both retrospectively and prospectively, they may potentially emulate clinical trials and observational studies, and allow for the adjustment for important prognostic factors and confounders. However, large representative databases for such analyses are available only in a few countries [16]. Database analyses are also limited by various factors including the lack of random assignments, lack of assurance of treatment adherence to the medication, lack of assessments based on specialized clinical instruments (e.g., disease specific rating scales), or detailed characterization of general physical health, etc.

To the best of our knowledge only one meta-analysis has been conducted so far to summarize empirical evidence, which is currently available from such data (using a real-world effectiveness endpoint which was time to all-cause medication discontinuation) in individual studies [17]. This meta-analysis focused on both observational studies and RCTs, but it compared only a single AP (olanzapine) with a limited set of APs. It should also be noted here that a comprehensive meta-analysis would not have been possible earlier due to the lack of availability of sufficient empirical data; with accumulating evidence this has now become possible. One major reason for the current lack of meta-analysis of real-world data is that the sample size in these studies is typically rather large, which appears to obviate the reason for a meta-analytic summary. Nonetheless, meta-analysis for real-world studies (RWS) can still be an important tool to synthesize the evidence and to assess the consistency and reliability of data that come from many samples or even populations, which can be clinically rather heterogeneous.

Thus, one of our principal goals in this investigation was to address the relevance of data from RCTs in schizophrenia (i.e., their predictive value) for the clinical practice. This may or may not support how regulatory agencies specify clinical design requirements in order to obtain clinically generalizable data (e.g., in terms of inclusion and exclusion criteria, sample size, endpoint definition, length of treatment, etc). The clarification of this question is of great importance from societal and individual patient perspective as well. In particular, doctors want to provide, and patients want to receive the best available treatment; the exposure of patients to an ineffective medication may result in avoidable suffering, healthcare costs and burden for the society.

Based on the considerations mentioned above our aim in this study was (1) to conduct a meta-analysis of data obtained from real-world settings; and (2) to compare the results of this meta-analysis with previously published meta-analyses of randomized controlled trials (RCTmeta) implementing head-to-head comparisons of antipsychotic treatments in schizophrenia.

To accomplish the above goals, in the current study we examined APs approved for the treatment of schizophrenia. We selected 8 APs: the first-generation AP haloperidol (date of approval (DOA) in Belgium = 1959 [18], and 1967 in the USA [19]); two early second-generation antipsychotics clozapine (DOA in the USA = 1989 [20], and in Switzerland and Austria 1972 [21]), and amisulpride (DOA in France = 1986 [22], and in the United Kingdom 1998 [22]); four new second-generation antipsychotics including risperidone (DOA in USA = 1993 [20]), olanzapine (DOA in USA = 1996 [20]), quetiapine (DOA in USA = 1997 [20]) and aripiprazole (DOA in USA = 2002 [20]), and finally the first second-generation long-acting injectable risperidone (DOA in USA = 2003 [20]). The reason for focusing on this group of medications was that they have been widely used for the treatment of schizophrenia across various countries, providing sufficient data for long-term follow-up in real-world settings (as the last marketing approval in the selected set of APs took place >15 years ago), and they are included in meta-analytic summaries yielding sufficient empirical data for our study. All-cause treatment discontinuation has been selected as an endpoint in this meta-analysis, since it has been found useful in treatment research as “…a clinically meaningful outcome that reflects the input of both the patient and the clinician” [23]. The adoption of all-cause treatment discontinuation as one of the endpoints in future RCTs may help better translate and back-translate treatment data between clinical trials and clinical practice.

## Methods

### Study endpoint

For real-world studies we focused on relative risk (RR) of all-cause treatment discontinuation due to any reason, adjusted for confounders in the original articles, as a principal measure of effectiveness in real-world environment. For RCTs we used all-cause discontinuation due to any reason or, in case this measure was unavailable, the drop out from the clinical trial was investigated as a surrogate measure. In case no data on relative risk was available in a paper, we used the odds ratio or the hazard ratio. If none of the above measures were available, the study was not included in our meta-analysis.

### Included publications

The data source for the selection was the Pubmed database. The queries described below for selection were run on 25th of April 2020 without any limitation to the date of publication. We only focused on studies published in English.

### Selection of real-world studies (RWS)

The selection of publications for the real-world dataset was conducted in two stages.

At stage 1, we applied a priori defined search criteria for the identification of potentially relevant articles. The search criteria were organized according to the following three queries (Q1–Q3):

- Q1: antipsychotic*[Title/Abstract] AND ((real*[Title/Abstract] AND world*[Title/Abstract]) OR nationw*[Title/Abstract]) AND schizophren*[Title/Abstract] AND (effectiv*[Title/Abstract] OR discont*[Title/Abstract])
- Q2: schizophren*[Title/Abstract] AND discont*[Title/Abstract] AND observational[Title/Abstract]
- Q3: schizophren*[Title/Abstract] AND discont*[Title/Abstract] AND claim*[Title/Abstract]

The above queries yielded a total of 135, 69, and 36 articles, respectively, for the Q1, Q2, and Q3 queries. After merging the query results with the logical OR connection and omitting the duplicate entries, we obtained 224 unique articles. The dates when the articles were entered in Pubmed ranged from 01 March 1980 to 22 January 2020.

**Fig. 1:**
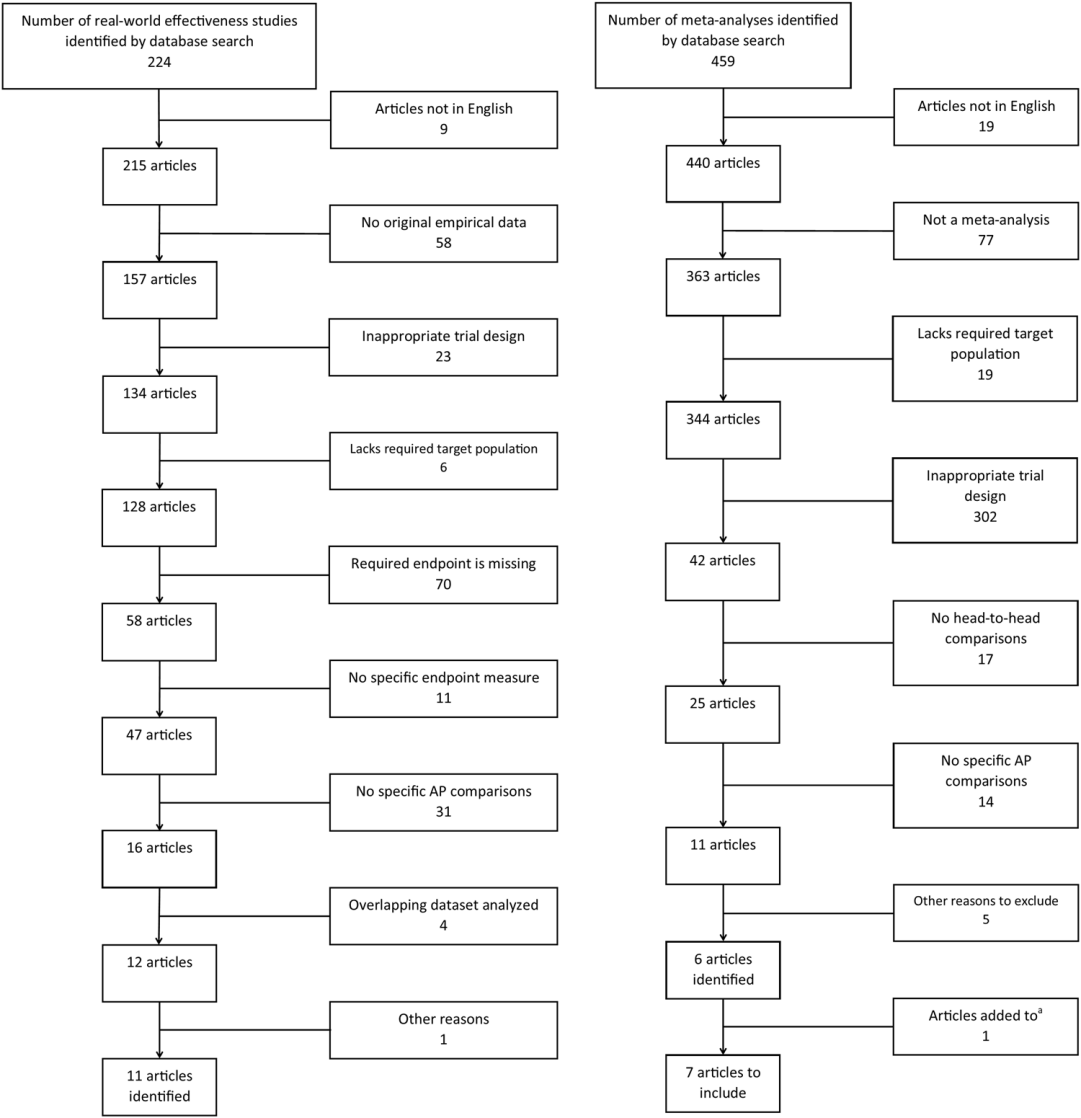
Flow chart of selection process of real-world effectiveness studies (left panel), and previously published meta-analyses based on randomized clinical trials (right panel). ^a^Article was identified during the review of selected papers: Sampson S, Hosalli P, Furtado VA, Davis JM; Risperidone (depot) for schizophrenia (Review); Cochrane Database Syst Rev 2016.

At stage 2, we implemented additional selection criteria in a hierarchical order to identify the final set of relevant publications. The selection criteria were employed using the full text papers (instead of the titles and abstracts) of the relevant publications. The selection process and the resultant set of publications is depicted in the flow chart shown in Fig. 1 (left panel). For further details of the selection process, please see Online Supplementary Information (Appendix 1). As a result of the selection process, we identified a total of 11 publications for the inclusion in the final set for our meta-analysis [16, 24–33].

As no direct head-to-head comparisons of monotherapies were published in the paper of Katona et al. [29], for the purpose of current investigation we used the relevant unpublished results available from the original analysis.

### Selection of meta-analyses of RCTs

Similar to the selection process of RWS, the selection of publications for the meta-analytic dataset also included two stages.

At stage 1, we applied search criteria for the identification of relevant publications. The search criteria were organized according to the following query.

- schizophren*[Title/Abstract] AND antipsychotic*[Title/Abstract] AND meta-analysis[Title/Abstract] AND (clinical[Title/Abstract] OR randomi*[Title/Abstract]) AND (trial*[Title/Abstract] OR study[Title/Abstract] OR studies[Title/Abstract])

The above query resulted in a total of 459 non-duplicate publications.

At Stage 2, we used additional selection criteria on the basis of the full text of the relevant publications. The selection process and the resultant set of publications is depicted in the flow chart shown in Fig. 1 (right panel). For further details of selection process, please see Online Supplementary Information (Appendix 2). As a result of the selection process, we identified six publications [17, 34–38], and our further literature review resulted in one more publication [39]. Hence, the set of publications that we used for the meta-analysis included a total of seven publications.

We note that during the selection for both RWS and meta-analytic summaries, the principal author identified the publications for inclusion; two of the authors (LK and PC) reviewed the results; in case of any discrepancy they had a discussion to achieve resolution.

### Statistical methods

#### Statistical model

The pooled effect size for the relative risk of all-cause treatment discontinuation due to any reason for each of compared AP pairs was estimated using normal mixture model with random effect for meta-analysis. On the basis of individual publications, we used the estimated relative risks and their standard errors (SE) as input data for the meta-analysis. All analyses were conducted using the SAS statistical software version 9.4 (SAS Institute, Inc., Cary, NC). For further details of statistical model, please see Online Supplementary Information (Appendix 3).

#### Statistical procedures

Since our investigation is not a review of the literature but a meta-analysis, we did not include those AP pairs in the analyses which were compared in only one real-world study.

Thus, our meta-analysis of RWS studies was based on AP comparisons where at least two publications were available with respect to a given pair-wise comparison. Moreover, in order to take the weight of the evidence into consideration, we also separately examined AP comparisons where three or more individual study data were available for the analysis. We examined whether statistically conclusive evidence (*p* < 0.05 for the pooled meta-analytic estimate) was available for a given AP comparison, and further investigated whether this evidence was present consistently across all individual studies. Consistency was defined as the individual study outcomes pointing always in the same direction in terms of their estimated effect size. Thus, we classified AP comparisons into three classes including study outcomes as follows:

- statistically conclusive and consistent;
- statistically conclusive but inconsistent;
- neither statistically conclusive nor consistent.

We note that for comparisons which included only two RWS, we provide the results in the Online Supplementary Information (Appendix 9, Part 2).

Since for the majority of RWS and RCTmeta comparison only one or two meta-analytic summaries were available we selected one primary and, whenever available, one secondary analysis as a benchmark for further comparisons. To consider a meta-analysis as an RCTmeta benchmark, we reviewed all the RCTs included in the meta-analysis. If there was a significant overlap between the RCTs, i.e., the majority of trials was included in both meta-analyses, we selected the meta-analyses which (1) was more recently published and/or (2) involved a larger number of RCTs. For those comparisons where more than one meta-analysis was available, we identified one primary and one secondary benchmark. To learn more about the selection process, please see Online Supplementary Information (eTable 1, Appendix 4).

**Table 1:** Descriptive characteristics of real-world studies selected for meta-analysis.

First author and year of publication	Title	Study design	Full list of individual APs (Involved in head-to-head comparisons) in the paper	Country where the Study was conducted	Follow-up period (months)	Selected APs involved in this meta-analysis	Control APs	Number of patients	Mean age	Percentage of male
Cooper et al. [26], 2005	Ambulatory use of olanzapine and risperidone: a population-based study on persistence and the use of concomitant therapy in the treatment of schizophrenia	Population-based cohort study, Quebec health insurance databases, 01 Jan 1997 to 31 Aug 1999 (2.5 years)	RIS, OLA	Canada	12	OLA	OLA	3687	NA	59.5
						RIS		2718	NA	54
Ascher-Svanum et al. [24], 2006	Time to discontinuation of atypical versus typical antipsychotics in the naturalistic treatment of schizophrenia﻿	Observational, non-randomized, multisite, prospective, naturalistic study, Jul 1997 to Sep 2003 (3 years)	CLO, OLA, RIS, QUE, ZIP, PER, HAL + AC	Various areas in the US	12	CLO	HAL + AC	114	37.3	57
						HAL + AC		114	38.2	57
						OLA		465	41.6	60.2
						QUE		178	39.6	47.2
						RIS		350	40.3	54.6
Tiihonen et al. [31], 2006	Effectiveness of antipsychotic treatments in a nationwide cohort of patients in community care after first hospitalization due to schizophrenia and schizoaffective disorder: observational follow-up study	Prospective cohort study using national central registers, 1995 to 2001 (7 years)	CLO, PER LAI, OLA, RIS, CHLM, CHLT, THI, PER, HAL, LEV	Finland	43.2 (mean)	CLO	HAL	150	27.4	NA
						HAL		37	31.5	NA
						OLA		197	28.6	NA
						RIS		240	30.7	NA
Haro et al. [28], 2007	Three-year antipsychotic effectiveness in the outpatient care of schizophrenia: observational versus randomized studies results﻿	Prospective observational longitudinal study, inclusion period Nov 2000 to Dec 2001 (follow-up period 3 years)	OLA, RIS, QUE, AMI, CLO	Denmark, France, Germany, Greece, Ireland, Italy, Netherlands, Portugal, Spain and the UK	36	AMI	OLA	256	38.9	54.1
						CLO		274	36.5	62.3
						OLA		4247	39.7	57.8
						QUE		583	40.5	51.6
						RIS		1549	39.6	57.7
Kilzieh et al. [30], 2008	Time to discontinuation and self-discontinuation of olanzapine and risperidone in patients with schizophrenia in a naturalistic outpatient setting	Retrospective study, electronic medical records database at a Veterans Affairs Medical Center, Jan 1999 to Dec 2000 (2 years)	RIS, OLA	US, Veteran Administrations Data	NA	OLA	OLA	221	NA	NA
						RIS		274	NA	NA
Dossenbach et al. [27], 2008	Long-term antipsychotic monotherapy for schizophrenia: disease burden and comparative outcomes for patients treated with olanzapine, quetiapine, risperidone, or haloperidol monotherapy in a pan-continental observational study	Prospective observational longitudinal study, inclusion period Nov 2000 to Dec 2001 (follow-up period 3 years)	HAL, OLA, QUE, RIS	Twenty-seven countries across four continents	36	HAL	OLA	189	35.1	52.9
						OLA		2641	34.8	54.8
						QUE		142	35.6	45.4
						RIS		863	36	51.9
Tiihonen et al. [32], 2011	A nationwide cohort study of oral and depot antipsychotics after first hospitalization for schizophrenia	Nationwide cohort study, national databases, 2000–2007 (8 years)	HAL LAI, OLA, CLO, RIS LAI, QUE, PER LAI, ZUC LAI, RIS, ZUC, HAL, PER	Finland	24 (mean)	CLO	RIS	188	NA	NA
						HAL		9	NA	NA
						OLA		389	NA	NA
						QUE		80	NA	NA
						RIS LAI		51	NA	NA
						RIS		411	NA	NA
Bitter et al. [25], 2013	Comparative effectiveness of depot and oral second generation antipsychotic drugs in schizophrenia: a nationwide study in Hungary	Nationwide, full-population based, insurance databases, 01 Jan 2006 to 30 Jun 2008 (2.5 years)	AMI, ARI, CLO, OLA, QUE, RIS, RIS LAI, ZIP	Hungary	12	AMI	Pairwise	920	47.01	38.2
						ARI		601	43.26	37.6
						CLO		790	48.31	46.8
						OLA		1633	46.27	41.3
						QUE		1578	49.72	34.2
						RIS LAI		1095	45.62	43.6
						RIS		2480	50.63	40.2
Katona et al. [29], 2014	Real-world effectiveness of antipsychotic monotherapy vs. polypharmacy in schizophrenia: to switch or to combine? A nationwide study in Hungary	Nationwide population-based study, insurance databases, Jan 2007 to Dec 2009 (3 years)	AMI, ARI, CLO, FLP LAI, FLPH LAI, HAL, HAL LAI, OLA, QUE, RIS, RIS LAI, ZIP, ZUC, ZUC LAI	Hungary	12	AMI	pairwise	420	46.8	36
						ARI		523	43.3	35
						CLO		217	46.9	45
						HAL		77	54.3	38
						OLA		794	47.8	39
						QUE		1311	52.3	35
						RIS LAI		352	47.7	41
						RIS		916	51.1	39
Tiihonen et al. [33], 2017	Real-world effectiveness of antipsychotic treatments in a Nationwide Cohort of 29823 Patients With Schizophrenia	Prospectively gathered nationwide databases, 01 Jul 2006 to 31 Dec 2013 (7.5 years)	ARI, CLO, FLU, FLP LAI, FLPH LAI, HAL, HAL LAI, LEV, OLA, OLA LAI, PAL LAI, PER, PER LAI, QUE, RIS, RIS LAI, ZUC, ZUC LAI	Sweden	68.4 (mean)	ARI	OLA	7300	NA	NA
						CLO		5191	NA	NA
						HAL		3348	NA	NA
						OLA		11730	NA	NA
						QUE		6004	NA	NA
						RIS LAI		3021	NA	NA
						RIS		7016	NA	NA
Takács et al. [16], 2019	Comparative effectiveness of second generation long-acting injectable antipsychotics based on nationwide database research in Hungary	Nationwide population-based study, insurance databases, 01 Jan 2012 to 31 Dec 2013 (2 years)	AMI, ARI, CLO, OLA, OLA LAI, PAL, PAL LAI, QUE, RIS, RIS LAI (comparators were: OLA LAI, PAL LAI, RIS LAI)	Hungary	24	AMI	RIS LAI	736	49.3	38
						ARI		921	43.7	38
						CLO		713	49.2	44
						OLA		2650	47.9	44
						QUE		2229	53.6	34
						RIS LAI		869	46.7	48
						RIS		2096	52.1	40

Owing to the within-subject approach applied as the primary analysis in Tiihonen et al. [33] study, no disjunct patient groups were formed, and therefore no unique number of patients was available for the individual treatments (as a given patient could have been subjected to various APs during the study period, i.e., may have been counted in multiple groups).

*AC* prophylactic anticholinergic agents, *AMI* amisulpride, *ARI* aripiprazole, *CHLM* chlorpromazine, *CHLT* chlorprothixene, *CLO* clozapine, *FLP* flupentixol, *FLPH* fluphenazine, *HAL* haloperidol, *LEV* levomepromazine, *OLA* olanzapine, *NA* data was not available, *PAL* paliperidone, *PER* perphenazine, *QUE* quetiapine, *RIS* risperidone, *RIS* LAI long acting injectable of risperidone, *THI* thioridazine, *ZIP* ziprasidone, *ZUC* zuclopenthixol.

In the final step in our analysis, we examined the congruency of the RWS estimates with RCTmeta benchmark(s). Congruency was defined as the correspondence between the sign of the pooled effect size estimate from the RWS and the benchmark RCTmeta analysis. Our presentation of the pertinent results was organized based on the presence/absence of statistically conclusive results in the RWS studies. Accordingly, we classified the comparison outcomes as follows:

- RWS statistically conclusive and showing congruency with RCTmetas;
- RWS statistically inconclusive but showing congruency with RCTmetas;
- RWS statistically conclusive with incongruence with RCTmetas;
- RWS statistically inconclusive and incongruent with RCTmetas.

We note that a number of comparisons for congruency could not be carried out in this analysis since no RCTmetas were available in the literature for the given AP comparisons we examined.

We also note that in order to increase clarity for the presentation of AP comparisons we used alphabetical order; therefore, whenever needed, we reciprocated the value that was provided in the original paper.

## Results

### Analyses of RWS

#### Descriptive statistics

We included a total of 11 studies in our meta-analysis based on the real-world data. Table 1 provides a brief description of the included studies. With respect to study design, 8 of 11 were based on database analysis using electronic medical/health insurance records and three of them represented observational studies. The list of the eight selected APs we investigated were as follows: amisulpride oral (AMI), aripiprazole oral (ARI), clozapine oral (CLO), haloperidol oral (HAL), olanzapine oral (OLA), quetiapine oral (QUE), risperidone oral (RIS), and risperidone LAI (RIS LAI).

Table 1 for each study displays both the full list of APs investigated in a given study, and the ones from the set of the 8 APs we focused on (listed above). In the next column (termed as “Control APs” in the table), we depicted those APs which were used as comparators in the original study. There were two studies which had more than one comparator in their respective pair-wise comparisons [25, 29]. As to the follow-up period of selected studies, the minimum duration was 12 months (*n* = 4), while there were studies which lasted 3 years or longer (*n* = 4). In one article, the length of follow-up period was not reported [30]. For the overwhelming majority of studies, a unique number of patients assigned to a given study medication was provided for each study arm; there was one study [33] where a within-subject approach was applied and no disjunct patient groups were formed, thereby no unique number of patients was available for the individual treatments. For the ten studies where the data in terms of unique treatment assignments were available, the total number of patients were the following: AMI = 2332, ARI = 2045, CLO = 2446, HAL = 426, OLA = 16924, QUE = 6101, RIS = 11897, and RIS LAI = 2367. Regarding basic demographic data, the mean age and/or gender distributions were published in eight papers. The mean and standard deviation of patients’ age (in years) for the selected APs were as follows: AMI = 45.5 (4.54), ARI = 43.4 (0.24), CLO = 40.9 (8.65), HAL = 39.8 (10.06), OLA = 41.0 (7.26), QUE = 45.2 (7.58), RIS = 42.9 (8.42), and RIS LAI = 46.7 (1.04). Furthermore, the percentage of male patients for each of the selected APs were as follows: AMI = 40.4%, ARI = 43.8%, CLO = 48.5%, HAL = 54.0%, OLA = 49.2%, QUE = 47.3%, RIS = 49.2%, and RIS LAI = 43.8%.

Raw data sources of the current meta-analysis in the original publications are depicted in Online Supplementary Information (eTable 2, Appendix 5).

### Meta-analyses of RWS

#### Pooled results with two or more RWS datasets available

Here we investigated only those AP comparisons where at least two real-world results were available for the given pair-wise comparisons. The 8 APs can potentially yield 28 unique pair-wise comparisons. Based on the criterion of the availability of multiple studies for the pair-wise comparisons, out of the 28 comparisons we identified a total of 25 AP comparisons that could be subjected to the meta-analysis.

**Fig. 2:**
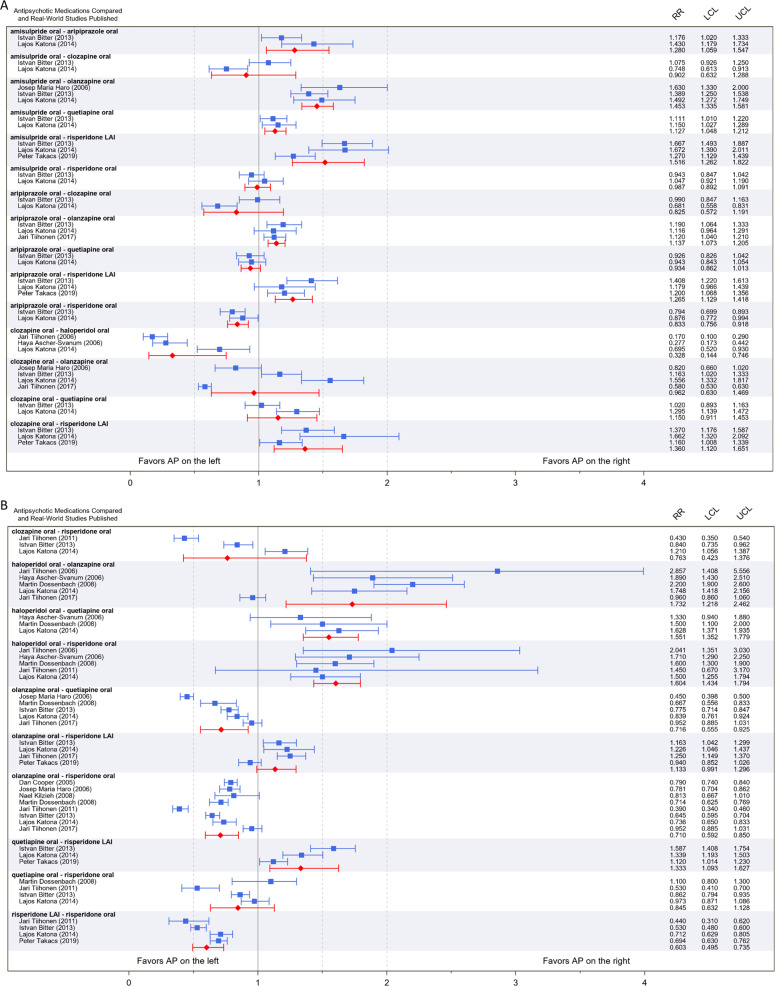
Treatment discontinuation due to any reason. Results of individual studies included in the meta-analysis along with pooled meta-analytic estimates based on random effect model. The figure provides the results for the 25 individual pair-wise comparisons. The results of pair-wise comparison are arranged in alphabetical order. **A** Results for the first set of comparisons (*n* = 15); **B** Results for the second set of comparisons (*n* = 10). Relative Risk of discontinuation for the first and second APs is indicated as a value of <1 or >1, depending on whether the first or the second AP in the pair has superior or inferior efficacy, respectively. For example, in the clozapine vs. haloperidol pairwise comparison, clozapine was found to be superior over haloperidol as our pooled estimate was 0.33, while in the amisulpride vs. olanzapine comparison, olanzapine showed superiority over amisulpride with an RR of 1.45. For the graphical illustration, the UCL value (5.556) was truncated at 4 in the case of the haloperidol oral–olanzapine oral pair-wise comparison from the Jari Tiihonen (2006) study. *RR* relative risk, *LCL* lower confidence limit, *UCL* upper confidence limit. Blue The results of individual real-world studies, Red Pooled estimates of current meta-analysis.

In 16 (64%) of the 25 individual paired comparisons our meta-analysis showed a significant difference between the two treatments. In terms of the magnitude of effect sizes, a large effect size was observed in one pair-wise comparison (CLO-HAL, RR = 0.33). In five of the remaining 24 comparisons the effect sizes were in the medium range (RR [or its reciprocal value] between 1.5 and 2). Figure 2A, B provide a graphical illustration and detailed numerical results of the individual pair-wise comparisons.

Due to the alphabetical order of pair-wise comparisons, in Fig. 2A, B we indicate by an asterisk (*) whenever a reciprocal value of RR was used in the presentation in this paragraph. The rationale for this was to present the relative risks of the effectiveness of a particular AP in a consistent way compared to its comparators. Overall, OLA showed superiority in 5 of 7 comparisons including AMI, ARI, HAL, QUE, and RIS with reducing the risk of all-cause discontinuation to 0.69*, 0.88*, 0.58*, 0.72, and 0.71, respectively, while for the rest of the comparators (CLO, RIS LAI) there was no significant difference. RIS LAI was superior over five APs in six available comparisons including AMI, ARI, CLO, QUE, and RIS with a risk reduction to 0.66*, 0.79*, 0.74*, 0.75*, and 0.6, respectively, while for one comparator (OLA) there was no significant difference. ARI had superior efficacy in two of six available comparisons (RR for AMI = 0.78, RIS = 0.83) with reducing the risk of all-cause discontinuation (while it was inferior to OLA and RIS LAI). QUE was superior in two of seven available AP comparisons (RR for AMI = 0.88*, HAL = 0.64*) with reducing the risk of all-cause discontinuation (while it was inferior to OLA and RIS LAI). CLO and RIS were superior, respectively, to one comparator (CLO vs. HAL RR = 0.33, RIS vs. HAL RR = 0.65*).

#### Pooled results with three or more RWS datasets available

There were 17 out of 25 comparisons with three or more real-world studies included. We investigated whether these groups (i.e., AP pairs compared) yielded homogenous or heterogenous results in terms of the estimated effect sizes. We considered a group homogenous if the majority of the input data (individual study level estimates of all the relative risks within a group) from real-word studies were significantly less than 1; or greater than 1; or not differentiated from 1. There were 12 AP pairs (70.6%) where the input data can be considered homogenous: AMI-OLA, AMI-RIS LAI, ARI-OLA, ARI-RIS LAI, CLO-HAL, CLO-RIS LAI, HAL-QUE, HAL-RIS, OLA-QUE, OLA-RIS, QUE-RIS LAI, RIS LAI-RIS. We identified five groups (29.4%) where the input data were heterogenous: CLO-OLA, CLO-RIS, HAL-OLA, OLA-RIS LAI, QUE-RIS.

Specifically, out of the 17 pair-wise comparisons we found that 12 (70.6%) provided both statistically conclusive and consistent evidence for the relative superiority of the respective APs in the comparisons. One of the comparisons (5.9%) yielded statistically conclusive but inconsistent result across individual studies. The remaining four comparisons (23.5%) were both statistically inconclusive and inconsistent among each other. Please note that the proportion of AP comparison which relied on more than three individual studies did not differ across the three categories. Specifically, considering the group with statistically conclusive and consistent results there was one comparison which included eight individual studies; there were two comparisons relying on five studies; one based on four studies; and eight on three studies. For the group of statistically conclusive but inconsistent results there was one comparison which was based on five individual studies. Finally, in the group of statistically inconclusive and inconsistent results there were three comparisons which included four individual studies; one based on three studies.

With respect to the both statistically conclusive and consistent outcomes we found the following order of superiority (indicated by “>” greater sign) in terms of relative risk of all-cause discontinuation for the AP pairs in the individual pair-wise comparisons: OLA > RIS, OLA > QUE, HAL < RIS, RIS LAI > RIS, AMI < OLA, ARI < OLA, ARI < RIS LAI, CLO > HAL, AMI < RIS LAI, CLO < RIS LAI, HAL < QUE, and QUE < RIS LAI. For the group with statistically conclusive but inconsistent results we found the following order of superiority: HAL < OLA. The remaining four pair-wise comparisons with statistically inconclusive and inconsistent results were as follows: CLO-OLA, QUE-RIS, OLA-RIS LAI, and CLO-RIS.

Detailed findings for each of the pair-wise comparisons according to above categories are provided in Online Supplementary Information (Appendix 6).

### Previously published meta-analyses based on RCTs (RCTmeta) as compared to meta-analytic results of RWS

#### Selection of primary and secondary benchmarks for comparisons with RWS

Our literature search yielded seven meta-analyses of RCTs. As described in the “Methods” section, we selected one primary and, whenever available, one secondary benchmark comparison for the RCTmetas (please see “Methods” section for details of the selection process). Overall, for the 17 AP RWS comparisons which had three or more results (of the total of 25 comparisons), we identified 13 relevant AP comparisons in previously published meta-analyses. For those comparisons where more than one RCTmetas were available we used both the primary and secondary benchmarks.

In Online Supplementary Information (eTable 3, Appendix 7) we provide basic descriptive statistics (i.e., the number of RCTs included) about prior meta-analyses based on randomized clinical trials.

#### Comparison of RWS with meta-analytic benchmarks from RCTs

The following part of this section is organized according to whether RWS provided conclusive evidence and/or the evidence was congruent with RCTmetas. Our results indicated that of nine RWS with statistically conclusive findings, the majority (*n* = 7; 77.8%) were congruent with RCTmetas. Of the three RWSs with statistically inconclusive findings, (*n* = 2; 66.7%) were congruent with the RCTmetas (see Online Supplementary Information (eTable 4, Appendix 8) for details).

The results of current study juxtaposed with the results of RCTmetas are presented in detail in a summary table (Table 2). In the following four sections of the Results, we rely on this table.

**Table 2:** Comparison of results from the meta-analysis of real-world studies that include three or more investigations, and from previously published meta-analyses based on RCTs.

Comparisons (three or more RWS in each)^a^	Number of RWS included	Findings conclusive (Yes/No)^b^	Individual study estimates are consistent (Yes/No)^c^	RR (95% CI) of our meta-analysis (based on RWS)^d^	RR (95% CI) of reference meta-analyses										Source of meta-analyses
					Evaluation of congruency										
					Primary benchmark	Secondary benchmark									
OLA-RIS	8	Yes	Yes	0.71 (0.59–0.85)	0.88 (0.83–0.93) congruent results	0.80 (0.71–0.90) congruent results									Primary: Kishimoto
															Secondary: Soares-Weiser
OLA-QUE	5	Yes	Yes	0.72 (0.56–0.92)	0.79 (0.71–0.89) congruent results										Primary: Kishimoto
HAL-RIS	5	Yes	Yes	1.60 (1.43–1.79)	0.87 (0.31–2.44) incongruent results										Primary: Samara
RIS LAI-RIS	4	Yes	Yes	0.60 (0.50–0.73)	1.17 (0.95–1.44) incongruent results	1.28 (0.92–1.79) incongruent results									Primary: Otsuzzi
															Secondary: Sampson
AMI-OLA	3	Yes	Yes	1.45 (1.34–1.58)	1.07 (0.91–1.27) congruent results										Primary: Kishimoto
ARI-OLA	3	Yes	Yes	1.14 (1.07–1.20)	1.17 (1.05–1.30) congruent results										Primary: Kishimoto
ARI-RIS LAI	3	Yes	Yes	1.26 (1.13–1.42)	1.20 (0.77–1.89) congruent results										Primary: Sampson
CLO-HAL	3	Yes	Yes	0.33 (0.14–0.75)	0.53 (0.29–1.12) congruent results										Primary: Samara
AMI-RIS LAI	3	Yes	Yes	1.52 (1.26–1.82)											
CLO-RIS LAI	3	Yes	Yes	1.36 (1.12–1.65)											
HAL-QUE	3	Yes	Yes	1.55 (1.35–1.78)											
QUE-RIS LAI	3	Yes	Yes	1.33 (1.09–1.63)											
HAL-OLA	5	Yes	No	1.73 (1.22–2.46)	1.54 (0.94–2.56) congruent results	1.40 (1.20–1.70) congruent results									Primary: Soares-Weiser
															Secondary: Beasley
CLO-OLA	4	No	No	0.96 (0.63–1.47)	1.01 (0.86–1.18) congruent results	1.05 (0.75–1.47) congruent results									Primary: Kishimoto
															Secondary: Soares-Weiser
QUE-RIS	4	No	No	0.84 (0.63–1.13)	1.07 (0.98–1.18) incongruent results										Primary: Kishimoto
OLA-RIS LAI	4	No	No	1.13 (0.99–1.30)											
CLO-RIS	3	No	No	0.76 (0.42–1.38)	0.74 (0.57–0.95) congruent results										Primary: Kishimoto

*AMI* amisulpride, *ARI* aripiprazole, *CI* confidence interval, *CLO* clozapine, *HAL* haloperidol, *OLA* olanzapine, *QUE* quetiapine, *RR* relative risk, *RIS* risperidone, *RIS LAI* long acting injectable of risperidone.

^a^APs underlined indicates superiority over their pairs compared to.

^b^It indicates statistically significant difference between the two APs (13/17 AP pairs).

^c^Point in the same direction.

^d^We identified 12 out of 17 comparisons with both RWS and RCT meta-analyses available.

#### AP comparisons of RWS with statistically conclusive (“significant”) results showing congruency with RCTmetas

OLA-RIS: Our meta-analysis yielded an RR of 0.71 (95% CI = 0.59-0.85) favoring OLA. We identified one RCTmeta for primary and one for secondary benchmark, respectively. These meta-analyses had both numerically and statistically congruent results with our estimate (RR = 0.88 (95% CI = 0.83–0.93); RR = 0.80 (95%CI = 0.71–0.90)).

OLA-QUE: The current meta-analysis resulted in an RR of 0.72 (95% CI = 0.56–0.92) favoring OLA. We identified one RCTmeta for primary benchmark, and none for secondary benchmark. The primary benchmark meta-analysis had both numerically and statistically congruent result with our estimate (RR = 0.79 (95% CI = 0.71–0.89)).

ARI-OLA: Our pooled estimate for RR was 1.14 (95% CI = 1.07–1.20) favoring OLA. We identified one primary and no secondary RCTmeta benchmark. The primary benchmark resulted in both numerically and statistically congruent result with our estimate (RR = 1.17 (95% CI = 1.05–1.30).

ARI-RIS LAI: Our meta-analysis yielded an RR of 1.26 (95% CI = 1.13–1.42) favoring RIS LAI. We found only one RCTmeta as benchmark which showed a numerically congruent result with our estimate (RR = 1.20 (95% CI = 0.77–1.89). However, the latter RCTmeta estimate did not reach statistical significance due to the low number of trials included in the meta-analysis (*N* = 2).

CLO-HAL: The current meta-analysis resulted in an RR of 0.33 (95% CI = 0.14–0.75) favoring CLO. We selected only one RCTmeta for benchmark, which had a numerically congruent result with our estimate (RR = 0.53 (95% CI = 0.29–1.12). The latter RCTmeta estimate was not statistically significant owing to the low number of trials included in the meta-analysis (*N* = 3).

AMI-OLA: Our pool estimate for RR was of 1.45 (95% CI = 1.34–1.58) favoring OLA. We identified only one RCTmeta for benchmark. This meta-analysis had numerically congruent result with our estimate in terms of direction (RR = 1.07 (95% CI = 0.91–1.27)). This RCTmeta estimate did not reach statistical significance due to the modest effect size (RR = 1.07).

HAL-OLA: Our meta-analysis provided an RR of 1.73 (95% CI = 1.22–2.46) favoring OLA. We identified one RCTmeta for primary and one for secondary benchmark, respectively. These meta-analyses had both numerically and statistically congruent results with our estimate (RR = 1.54 (95% CI = 0.94–2.56); RR = 1.40 (95% CI = 1.20–1.70)).

#### Comparisons of RWS with statistically inconclusive results showing congruency with RCTmetas

CLO-OLA: The current meta-analysis resulted in RR of 0.96 (95% CI = 0.63–1.47), showing a lack of difference. We identified one RCTmeta for primary and one for secondary benchmark, respectively. These meta-analyses had both numerically and statistically congruent results with our estimate (RR = 1.01 (95% CI = 0.86–1.18); RR = 1.05 (95% CI = 0.75–1.47)), with no difference between these two APs.

CLO-RIS: Our pooled estimate for RR was 0.76 (95% CI = 0.42–1.38) which failed to reach statistical significance. We found only one RCTmeta for benchmark, which had a numerically congruent result with our estimate (RR = 0.74 (95% CI = 0.57–0.95)).

**Fig. 3:**
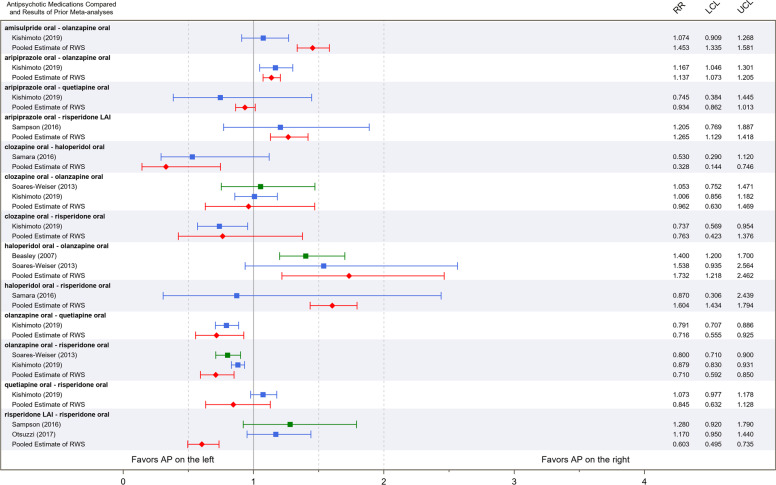
Effect size estimates from previously published meta-analyses of RCTs, and from current meta-analysis. Outcome measure: treatment discontinuation due to any reason Groups: primary benchmark (blue); secondary benchmark (green); and pooled estimates of current meta-analysis (red). Please note that for the comparison of aripiprazole-quetiapine the pooled estimate RWS is only based on two studies. *RR* relative risk, *LCL* lower confidence limit, *UCL* upper confidence limit. *Blue* The results of individual meta-analyses based on randomized controlled trials’ data considered as primary benchmark. *Green* The results of individual meta-analyses based on randomized controlled trials’ data considered as secondary benchmark. *Red* Pooled estimates of current meta-analysis.

ARI-QUE: The current meta-analysis resulted in RR of 0.93 (95% CI = 0.86–1.01), showing a lack of difference. We identified one RCTmeta as primary benchmark which showed congruent result with our estimate (RR = 0.75 (95% CI = 0.38–1.45)). However, we note that our estimate here was based on only two available RWS (this result is presented in Fig. 3 but not in Table 2).

#### Comparison of RWS with statistically conclusive results showing incongruence with RCTmetas

HAL-RIS: Our meta-analysis provided an RR estimate of 1.60 (95% CI = 1.43–1.79). We had only one RCTmeta for benchmark, which provided a numerically incongruent result with our estimate (RR = 0.87 (95% CI = 0.31–2.44)), but did not reach statistical significance.

RIS LAI-RIS: The current meta-analysis resulted in an RR of 0.60 (95% CI = 0.50–0.73). We identified one RCTmeta for primary and one for secondary benchmark, respectively. These RCTmetas yielded a numerical (but not statistically significant) advantage for RIS vs. RIS-LAI (RR = 1.17 (95% CI = 0.95–1.44); RR = 1.28 (95% CI = 0.92–1.79)), while the meta-analysis of RWS showed a statistically significant superiority for RIS-LAI over RIS.

#### Comparison of RWS with statistically inconclusive results showing incongruence with RCTmetas

QUE-RIS: Our meta-analysis resulted in an RR of 0.84 (95% CI = 0.63–1.13) which failed to reach statistical significance. We found only one RCTmeta for benchmark, which had a numerically incongruent result with our estimate (RR = 1.07 (95% CI = 0.98–1.18)).

#### No RCTmetas were available

There were five comparisons which we could not identify any RCTmetas for: AMI-RIS LAI, CLO-RIS LAI, HAL-QUE, QUE-RIS LAI, and OLA-RIS LAI.

## Discussion

The first objective of our study was to address an existing lack of information in the literature, namely the availability of the summary of evidence from real-world studies about the head-to-head comparisons of antipsychotic medications. This goal is clinically highly relevant since RCTs very rarely yield information for the comparative efficacy (or effectiveness) of an individual AP against its potential comparators used in clinical practice. To achieve our goal, we conducted a meta-analysis of eight APs (amisulpride, aripiprazole, clozapine, haloperidol, olanzapine, quetiapine, risperidone, and risperidone LAI). Based on the analysis of the literature, we found that published data were available for most of the comparisons (25 of 28 theoretically possible contrasts).

For the majority of comparisons based on three or more RWS, our results indicated that the real-world studies yielded statistically conclusive and, clinically even more importantly, consistent findings across the individual investigations. Indeed, out of 17 studies with sufficient empirical data (i.e., ≥3 RWS), the proportion of studies with both conclusive and consistent results was 70.6% (12 of 17). For those studies with statistically inconclusive and inconsistent results, several factors might have come into play, including the lack of true difference among medications in real-world settings, sampling variation, heterogeneity of study populations, study design, or confounding by indication due to differing clinical practices across countries (discussion of these studies is provided in Online Supplementary Information (Appendix 9)).

When we examined AP comparisons with at least two available studies, we found replicated evidence for certain AP comparisons. Specifically, OLA showed superiority in five of seven comparison APs including AMI, ARI, HAL, QUE, as well as RIS in terms of reducing the risk of all-cause discontinuation, and no difference against CLO and RIS LAI. RIS LAI was superior over five APs of six available comparisons including AMI, ARI, CLO, QUE, and RIS with a risk reduction (even though for AMI, ARI, CLO, QUE only one national database provided information for the analysis). QUE was superior at reducing the risk of all-cause discontinuation in two of seven available comparisons, one of which was against HAL, and the other one was AMI (with data available only from one national database).

Our second objective was to compare the real-world evidence with evidence from RCTs. This comparison is crucial from a clinical standpoint since RCTs are considered as providing the highest level of evidence for clinical practice in individual studies, yet the matching of the two types of evidence (real-world and RCT) is seldom accomplished in the literature. Our results showed that for the overwhelming majority of the comparisons where both real-world and RCTmeta were available (12 comparisons), there was a good congruency (75%, i.e., nine of 12) between the real-world and the RCT results. In addition, among the real-world studies with statistically conclusive results, most comparisons (77.7%, i.e., seven of nine) yielded results similar to those in RCT trials. Findings of the current study are therefore consistent with findings from Soares-Weiser et al.’s meta-analysis [17], which showed a good overall consistency between RCTs and observational studies based on a limited set of pair-wise comparisons (comparing olanzapine to six other APs). Specifically, the effect sizes were numerically similar between RCTs and observational studies in terms of their direction, apart from a single comparison (olanzapine vs. clozapine). For further details, please see Online Supplementary Information (eTable 5, Appendix 10).

Thus, our results support that randomized controlled trials, despite all of their limitations, provide evidence which is generalizable to real-world settings. These include that RCTs focus on special populations (e.g., exclude difficult to treat or violent subjects), apply a small sample size (typically less than 150 subjects per study arm), placebo control (as it is easier to demonstrate pivotal evidence for efficacy against placebo than an active comparator), have short duration (typically less than 24 weeks), very limited follow-up period (precluding the detection of late occurring drug effects), and lack of clinically highly relevant real-world endpoints as primary measures (e.g., hospitalization, treatment discontinuation). Altogether, this is an important finding from the perspective of the regulators, who strive to set up study specifications and guidelines in order to achieve the highest level of generalizability from the clinical trial domain to the actual clinical practice. These results are also encouraging for the clinical practice: the results of RCTs showed a good correspondence with those of real-world data from large healthcare datasets. Nonetheless, the predictive value of RCTs for real-world practice should regularly be assessed. Specifically, there still remains a need to examine the generalizability of the results of RCTs in long-term multi-arm non-randomized naturalistic studies or in analyses of healthcare databases.

As we expected, while head-to-head comparisons were more readily available in real-world studies, they were much less frequent in randomized clinical trials. Specifically, we found that only for 12 of 17 RWS comparisons with sufficient evidence (i.e., three or more real-world studies included) were data available from RCT meta-analyses; these constituted the set of pair-wise comparisons used for studying the congruency between RWS and RCT studies. Thus, our study provides empirical evidence that the RCT data adequately translate to clinical settings. Nevertheless, while our results show the generalizability of evidence with respect to a number of AP comparisons, they evidently do not pertain to AP comparisons with no available data from RCTs. Hence, the theoretical questions of how the RWS data would translate back to RCT settings, and whether the results for AP pairs with missing data in RCT settings would similarly generalize remain to be studied further.

Finally, we would like to note that there were three comparisons with incongruent results between RWS and RCTs, which included HAL-RIS, RIS LAI-RIS, and QUE-RIS. These may be resulted from several factors including the small sample size of the individual trials, as well as the heterogeneity of samples (see a discussion of this issue in Online Supplementary Information (Appendix 11).

### Limitations

Pair-wise meta-analyses have been criticized in the literature because they typically rely on data from one pair of individual treatment comparison. In the current real-world meta-analysis, however, this limitation was not present when we took the weight of the evidence into consideration and relied predominantly on individual AP comparisons where at least three or more studies provided data. The fact that we were able to use multiple studies for the same pair-wise comparisons obviates the problem of heterogeneity at pooling data across various studies which is present in the current practice in network meta-analysis.

We also note that some of the differences in findings across RWS may come from differences in the methodological approaches including study design used by the various groups of investigators involved in the analysis (e.g., varying selection criteria such as inclusion of patients with first hospitalization). An additional limitation of our comparison of RWS and RCT meta-analyses could be that the studies included into the two analyses may have relied on different patient cohorts since therapeutic guidelines and practices changed over time with the introduction of newly approved APs to the market. However, an overview of publication dates and entry time window (2005–2019) for the two types of studies (i.e., RWS and RCT meta-analyses) reveals that they covered similar time periods, when the new second-generation antipsychotics were widely available on the market for treatment.

Furthermore, our analyses were limited by the fact that some of the basic descriptive demographic information was not available in five of the 11 RWS. Therefore, these data could not be included in our analysis in order to identify the sources that might have caused heterogeneity in the estimates of RWS. The availability of such data for the purpose of research synthesis including meta-analysis underlines the importance of reporting this information. In addition, the inconsistent findings in RCTs versus RWS about the comparative effectiveness of oral versus LAI formulations of second-generation APs have been addressed earlier: “LAIs are thought to be better via improved adherence, not via intrinsically better efficacy. Therefore, it is unclear whether LAIs were not superior because compliance with oral APs was good enough in the context of RCTs.” [40]. Thus, these findings may be explained by different rates of adherence in pivotal RCTs versus RWS.

Finally, our findings from the real-world studies that rely mostly on a limited set of countries (e.g., Scandinavian countries and Hungary) can be influenced by regional differences [41], therefore we cannot be certain that the results would generalize to most countries and regions of the world. We note, however, that some of RWS cover broad geographical regions that include the four continents or multiple European countries, which can add support to the notion of the broader generalizability of the results. Furthermore, the RWS show an imbalance in terms of the representations of the countries as some of the countries provided disproportionally more impute data for our analysis. For example, with respect to the comparisons with data from three or more RWS, there were some important comparisons where data was only available from one national health database (for amisulpride, aripiprazole and quetiapine vs risperidone LAI). Additionally, the RWS from various countries that we used in our study can be influenced by between country or regional differences in therapeutic practices, which determine medication assignments to various APs (e.g., due to different national guidelines). Nonetheless, we conclude that our results show a rather remarkable consistency in individual pairs of AP comparisons across studies, as well as a good congruency with the results of RCT meta-analyses.

## Conclusions

The principal conclusion of the current study is that results of RCTs evidence a good congruency with those of the real-world studies. Moreover, the results of our analyses, taken together, hold out a promise that the findings of the RWS analysis would provide useful and much needed information for clinicians for everyday practice. Importantly, the findings of RWSs that pertain to comparisons of antipsychotic medications not yet subjected to clinical testing in RCTs are essential for three major reasons. First, they may provide putative guidance for practicing clinicians for situations where no evidence is available from double-blind clinical trials. Second, they can also provide specific testable hypotheses for clinically important questions in future clinical trials. Third, data from RWS analysis can provide essential information for regulators with respect to design requirements for future studies. For example, in terms of effect size criteria, RWS data would be essential to demonstrate superiority or equivalence of a new antipsychotic under development in the context of differences among currently available antipsychotic medications. While we focused on APs, we think that our results may have relevance to other therapeutic areas (e.g., diabetes, high blood pressure, etc.) since the use of APs can be considered as a prototype for medical conditions, where long term therapy presents a major challenge in patients living with chronic lifelong conditions. However, further studies are needed in other areas of indications in order to confirm the generalizability of our findings.

## Supplementary information

Online Supplementary Information
