# Supplementary material for: A meta-analysis of effectiveness of real-world studies of antipsychotics in schizophrenia: Are the results consistent with the findings of randomized controlled trials?
Source: Transl Psychiatry. 2021 Oct 6;11:510. doi: 10.1038/s41398-021-01636-9 (PMC8494924; doi:10.1038/s41398-021-01636-9)
Supplement: Supplementary file 1 — Online Supplementary Information [file 41398_2021_1636_MOESM1_ESM.docx]

Katona *et. al.* (2021): A meta-analysis of effectiveness of real-world studies of antipsychotics in schizophrenia: Are the results consistent with the findings of randomized controlled trials?

**Online Supplementary Information**

Table of Contents

[Appendix 1: Selection process of real-world studies (RWS) 2](#_Toc77176307)

[Appendix 2: Selection process of RCT meta-analyses 2](#_Toc77176308)

[Appendix 3: Statistical model of current investigation 3](#_Toc77176309)

[Appendix 4: Selection of previously published meta-analyses based on RCTs (RCTmeta) 4](#_Toc77176310)

[Appendix 5: Data sources of the current meta-analysis in the original publications (RWS) 8](#_Toc77176311)

[Appendix 6: Detailed results of current meta-analysis (RWS) 9](#_Toc77176312)

[Part 1: Comparisons with 3 or more RWS included (17 out of 25 comparisons) 9](#_Toc77176313)

[Part 2: Comparisons with only 2 RWS included (8 out of 25 comparisons) 11](#_Toc77176314)

[Appendix 7: Basic descriptive statistics of previously published meta-analyses (RCTmeta) 13](#_Toc77176315)

[Appendix 8: Comparison of RWS with meta-analytic benchmarks from RCTs (RCTmeta) 15](#_Toc77176316)

[Appendix 9: Discussion about comparisons with statistically conclusive but inconsistent results 15](#_Toc77176317)

[Appendix 10: Results from the meta-analysis of Soares-Weiser *et. al.* (2013) 16](#_Toc77176318)

[Appendix 11: RWS vs RCTs with incongruent results 16](#_Toc77176319)

[References 17](#_Toc77176320)

# Appendix 1: Selection process of real-world studies (RWS)

On the first level of selection shown in Figure 1 (left panel), we excluded 9 articles as they were published in a language other than English. Next, we excluded 58 papers which did not include relevant original empirical data published (i.e., they were a review, meta-analysis, letter, guideline, etc). Furthermore, there were 23 studies published with a design not eligible for the requirement of a real-world study setting (no specific APs, randomized clinical trial, survey, theoretical paper, dose reduction study, etc).

We excluded 6 articles due to the lack of required study population (the inclusion criterion of the study was not ‘schizophrenia or schizoaffective disorders’). A total of 70 publications were excluded which did not have the required endpoint (i.e., time to all-cause discontinuation due to any reason). There were 11 studies that we excluded since they did not use the specific endpoint measures (neither odds ratios nor hazard ratios were calculated). Due to a lack of specific AP comparisons, we excluded further 31 publications. Moreover, we did not include 4 papers with analyses based on overlapping datasets. Finally, we excluded one paper due to insufficient information to determine relevant summary statistical information on required endpoint^1^, listed as other reason. Hence, we identified a total of 11 publications for the inclusion in the final set for our meta-analysis.

# Appendix 2: Selection process of RCT meta-analyses

Out of the 459 papers selected by the automatic search shown in Figure 1 (right panel), we excluded 19 as they were written in a language other than English. On the next level, 77 articles were excluded as they were not meta-analyses. Further 19 were excluded because they lack the required study population (i.e., ‘schizophrenia or schizoaffective disorders’). Subsequently, 302 publications were excluded due to trial design features, which did not meet the requirements of the current investigation (was not a psychopharmacological study, e.g., Transcranial Magnetic Stimulation, ECT, physical exercise; had only specific safety measures (e.g. metabolic syndromes); was a network meta-analysis, as their results are not based on direct pair-wise comparisons from individual studies). Moreover, 17 meta-analyses were excluded because they did not include head-to-head comparisons of AP medications. Additionally, those meta-analyses were excluded since did not include the selected APs that we examined in our current analysis. Finally, 5 meta-analyses were excluded due to other reasons (overlapping datasets, no head-to-head comparison in specific APs, no relevant numerical results, excluded site data processed in the source meta-analysis). Thus, the set of publications that we used for the meta-analysis included a total of 7 publications.

# Appendix 3: Statistical model of current investigation

The pooled effect size for the relative risk of all-cause treatment discontinuation due to any reason for each of compared AP pairs was estimated using the random effect model for meta-analysis. The random effect model was chosen because heterogeneity, regardless of its statistical significance, is expected to be present across investigations that may have differences in their design and study populations. The analysis under heterogeneity was based on the DerSimonian and Laird (1986)^2^ normal mixture model, which considers each sample to be random sample from a normal population. We adopted the multivariate approach for our meta-analysis because it makes allowance for correlated effect sizes in the analyses^3^, and permits several effect size estimates from the same study. These conditions could in fact occur in our investigation since input data for the relative effectiveness for several AP pairs could be obtained from a single study.

Based on the individual study reports, we used the estimated relative risks and their standard errors (SE) as input data for the meta-analysis. In case the SE was not reported in the original publication, we used the 95% confidence intervals in order to calculate the standard error. Following the approach outlined by Van Houwelingen et al. (2002), based on the SE we computed the within-trial variance of the estimated relative risks; the inverse of the variance was applied as a weighting factor in determining the pooled estimates for the relative effectiveness of the compared AP pairs. We did not use the number of patients as weighting variable because there was one large study^4^ where a within-subject approach was used with no disjunct patient groups formed, and no unique number of patients was available for the individual treatments. Following Van Houwelingen et al. (2002)., we conducted the analyses by using the log-transformed values (i.e., log-relative risks and SEs). The pooled estimates (relative risk and SE) that were derived on the log-transformed scale were back transformed to the original scale, and thus were used for reporting purposes in the current investigation.

# Appendix 4: Selection of previously published meta-analyses based on RCTs (RCTmeta)

The table provided herein summarizes the selection process for the previously published meta-analyses, and the rationale for the designation of the primary and secondary benchmarks.

**eTable 1: Previously published meta-analyses based on RCTs^a^**

| **AP comparisons and individual RCTs published** | **Previously published meta-analyses based on RCTs (RCTmeta)** | | | | | | |
| --- | --- | --- | --- | --- | --- | --- | --- |
|  | **Beasley (2007)^5^** | **Kishimoto (2019)^6^** | **Krause (2018)^7^** | **Ostuzzi (2017)^8^** | **Samara (2016)^9^** | **Sampson (2016)^10^** | **Soares-Weiser (2013)^11^** |
| **AMI-OLA** |  | **3** |  |  |  |  | **3** |
| Kahn 2008 |  | 1 |  |  |  |  | 1 |
| Lecrubier 2006 |  | 1 |  |  |  |  | 1 |
| Mortimer 2004 |  | 1 |  |  |  |  |  |
| Mortimer 2007 |  |  |  |  |  |  | 1 |
| **ARI-OLA** |  | **8** |  |  |  |  | **2** |
| Chrzanowski 2006 |  | 1 |  |  |  |  |  |
| Crespo-Facorro 2014 |  | 1 |  |  |  |  |  |
| Fleischhacker 2009 |  | 1 |  |  |  |  | 1 |
| Kane 2009 |  | 1 |  |  |  |  | 1 |
| McQuade 2004 |  | 1 |  |  |  |  |  |
| NCT00802100 |  | 1 |  |  |  |  |  |
| Parabiaghi 2016 |  | 1 |  |  |  |  |  |
| Wani 2015 |  | 1 |  |  |  |  |  |
| **ARI-QUE** |  | **2** |  |  |  |  |  |
| Crespo-Facorro 2014 |  | 1 |  |  |  |  |  |
| Gaebel 2010; de Arce Cordón 2012 |  | 1 |  |  |  |  |  |
| **ARI-RIS LAI** |  |  |  |  |  | **2** |  |
| Gaebel 2010 |  |  |  |  |  | 1 |  |
| MacFadden 2010 |  |  |  |  |  | 1 |  |
| **CLO-HAL** |  |  |  |  | **4** |  |  |
| Buchanan 1998 |  |  |  |  | 1 |  |  |
| Kane 2001 |  |  |  |  | 1 |  |  |
| Rosenheck 1997 |  |  |  |  | 1 |  |  |
| Volavka 2002 |  |  |  |  | 1 |  |  |
| **CLO-OLA** | **3** | **4** |  |  | **7** |  | **4** |
| Bitter 2004 | 1 |  |  |  | 1 |  | 1 |
| Conley 2003 |  |  |  |  | 1 |  |  |
| McEvoy 2006 |  | 1 |  |  |  |  | 1 |
| Meltzer 2003 |  | 1 |  |  |  |  |  |
| Meltzer 2008 |  | 1 |  |  | 1 |  |  |
| Moresco 2004 |  |  |  |  | 1 |  |  |
| Naber 2005 |  | 1 |  |  | 1 |  |  |
| Tollefson 2001 | 1 |  |  |  | 1 |  | 1 |
| Volavka 2002 | 1 |  |  |  | 1 |  | 1 |
| **CLO-RIS** |  | **4** |  |  | **6** |  |  |
| Azorin 2001 |  |  |  |  | 1 |  |  |
| Bondolfi 1998 |  |  |  |  | 1 |  |  |
| Daniel 1996 |  |  |  |  | 1 |  |  |
| McEvoy 2006 |  | 1 |  |  |  |  |  |
| McGurk 2005 |  |  |  |  | 1 |  |  |
| NCT00573287 |  | 1 |  |  |  |  |  |
| Sanz-Fuentenebroet 2013 |  | 1 |  |  |  |  |  |
| Schooler 2016 |  | 1 |  |  |  |  |  |
| Volavka 2002 |  |  |  |  | 1 |  |  |
| Wahlbeck 2000 |  |  |  |  | 1 |  |  |
| **HAL-OLA** | **5** |  |  |  | **5** |  | **5** |
| Altamura 2002 |  |  |  |  | 1 |  |  |
| Breier 1999 |  |  |  |  | 1 |  |  |
| Buchanan 2005 |  |  |  |  | 1 |  |  |
| Kahn 2008 |  |  |  |  |  |  | 1 |
| Keefe 2006 | 1 |  |  |  |  |  | 1 |
| Lieberman 2003 |  |  |  |  |  |  | 1 |
| Lieberman 2005 | 1 |  |  |  |  |  |  |
| Purdon 2000 | 1 |  |  |  |  |  |  |
| Rosenheck 2003 | 1 |  |  |  |  |  | 1 |
| Smith 2001 |  |  |  |  | 1 |  |  |
| Volavka 2002 | 1 |  |  |  | 1 |  | 1 |
| **HAL-RIS** |  |  |  |  | **2** |  |  |
| Volavka 2002 |  |  |  |  | 1 |  |  |
| Wirshing 1999 |  |  |  |  | 1 |  |  |
| **OLA-QUE** |  | **8** |  |  |  |  | **6** |
| Deberdt 2008 |  | 1 |  |  |  |  | 1 |
| Kahn 2008 |  | 1 |  |  |  |  | 1 |
| Kinon 2006c |  | 1 |  |  |  |  | 1 |
| Lieberman 2005 |  | 1 |  |  |  |  | 1 |
| Lublin 2008 |  |  |  |  |  |  | 1 |
| McEvoy 2006 |  | 1 |  |  |  |  |  |
| McEvoy 2007 |  | 1 |  |  |  |  | 1 |
| Stroup 2006 |  | 1 |  |  |  |  |  |
| Stroup 2007 |  | 1 |  |  |  |  |  |
| **OLA-RIS** | **5** | **16** | **3** |  | **2** |  | **11** |
| Akerele 2007 |  |  |  |  |  |  | 1 |
| Alvarez 2006 |  | 1 |  |  |  |  |  |
| Apiquian 2003 |  |  |  |  |  |  | 1 |
| Chan 2010 |  | 1 |  |  |  |  |  |
| Chen 2012 |  |  |  |  | 1 |  |  |
| Ciudad 2006 |  |  |  |  |  |  | 1 |
| Crespo-Facorro 2012 |  | 1 |  |  |  |  |  |
| Feldman 2003 |  |  | 1 |  |  |  |  |
| Gureje 2003 | 1 |  |  |  |  |  | 1 |
| Jeste 2003 |  |  | 1 |  |  |  |  |
| Keefe 2006 | 1 | 1 |  |  |  |  | 1 |
| Kumar 2016 |  | 1 |  |  |  |  |  |
| Lieberman 2005 |  | 1 |  |  |  |  | 1 |
| Lublin 2008 |  |  |  |  |  |  | 1 |
| McEvoy 2006 |  | 1 |  |  |  |  |  |
| McEvoy 2007 |  | 1 |  |  |  |  | 1 |
| NCT00236379 |  | 1 |  |  |  |  |  |
| Noordsy et al59 |  | 1 |  |  |  |  |  |
| Purdon 2000 | 1 | 1 |  |  |  |  |  |
| Ritchie 2003 |  |  | 1 |  |  |  |  |
| Ritchie 2010 |  | 1 |  |  |  |  |  |
| Stroup 2006 |  | 1 |  |  |  |  |  |
| Stroup 2011 |  | 1 |  |  |  |  |  |
| Tran 1997 | 1 | 1 |  |  |  |  | 1 |
| Tunis 2007 |  | 1 |  |  |  |  |  |
| Volavka 2002 | 1 |  |  |  | 1 |  | 1 |
| Wang 2006 |  |  |  |  |  |  | 1 |
| **QUE-RIS** |  | **8** |  |  |  |  |  |
| Lieberman 2005 |  | 1 |  |  |  |  |  |
| Liu 2014 |  | 1 |  |  |  |  |  |
| McEvoy 2006 |  | 1 |  |  |  |  |  |
| McEvoy 2007 |  | 1 |  |  |  |  |  |
| Naber 2013; NCT00600756 57 |  | 1 |  |  |  |  |  |
| NCT00206102 |  | 1 |  |  |  |  |  |
| Stroup 2006 |  | 1 |  |  |  |  |  |
| Stroup 2007 |  | 1 |  |  |  |  |  |
| **RIS LAI-RIS** |  |  |  | **6** |  | **2** |  |
| Bai 2006 |  |  |  |  |  | 1 |  |
| Bai 2007 |  |  |  | 1 |  |  |  |
| Chue 2002 |  |  |  |  |  | 1 |  |
| Chue 2005 |  |  |  | 1 |  |  |  |
| Green 2015 |  |  |  | 1 |  |  |  |
| NCT00240708 |  |  |  | 1 |  |  |  |
| NCT00992407 |  |  |  | 1 |  |  |  |
| Subotnik 2015 |  |  |  | 1 |  |  |  |

^a^ Those studies are highlighted in yellow which were included in multiple meta-analyses.

AMI-OLA: We identified 2 RCTmetas, each of them has 3 RCTs included. There was an overlap between the two RCTmetas, as two RCTs were used in both of them. As a result of this, we only used Kishimoto et al.’s result as primary benchmark.

ARI-OLA: There were 2 RCTmetas available. Kishimoto et al. study included 8 RCTs, while Soares-Weiser et al. study only had 2 RCTs involved. We identified Kishimoto result as primary benchmark. As both of the RCTs included in Soares-Weiser meta-analysis were also analyzed in Kishimoto study, we decided not to use Soares-Weiser result as secondary benchmark.

CLO-OLA: There were 4 RCTmetas available. We decided not to use Samara’s because it was based on patients with treatment-resistant schizophrenia. We also excluded Beasley et al.’s study as all the RCTs included in their study were included in Soares-Weiser study. Finally, we used Kishimoto result as primary, and Soares-Weiser result as secondary benchmark.

CLO-RIS: We identified two RCTmetas, Kishimoto et al. and Samara et al. We decided not to use Samara’s publication because it was based on patients with treatment-resistant schizophrenia. We kept Kishimoto 2019 as primary benchmark.

HAL-OLA: There were 3 RCTmetas available. We decided not to use Samara’s publication because it was about treatment-resistant schizophrenia. Both Soares-Weiser et al. and Beasley et al. studies have a total of 5 RCTs included. There were 3 RCTs which were included in both RCTmetas. Based on our decision rule, outlined in the main text of the publication, we decided to use Soares-Weiser as primary, and Beasley as secondary benchmark.

OLA-QUE: We identified 2 RCTmetas. Kishimoto had 8, while Soares-Weiser included 6 RCTs in their analyses. As there was a significant overlap between the two RCTmetas with 5 RCTs, we decided to use only Kishimoto’s publication as primary benchmark.

OLA-RIS: There were a 5 RCTmetas available. We decided not to use Samara’s publication because it was based on patients with treatment-resistant schizophrenia. We also excluded Krause et al. study as it had only 3 RCTs included. As all RCTs that were included in Beasley analysis were all available in Soares-Weiser meta-analysis, we decided not to use Beasley study as reference. Finally, based on our decision rules outlined in the main paper we decided to keep Kishimoto as primary and Soares-Weiser as secondary benchmark.

RIS LAI-RIS: We identified 2 RCTmetas. We decided to include Otsuzzi *et. al.* study with 6 RCTs as primary benchmark, and Sampson et al. study which only had 2 RCTs as secondary benchmark.

There were five AP comparisons with one RCTmeta:

- ARI-QUE: Kishimoto et al. with 2 RCTs
- ARI-RIS LAI: Sampson et al. with 2 RCTs
- CLO-HAL: Samara et al. with 4 RCTs*
- HAL-RIS: Samara et al. with 2 RCTs*
- QUE-RIS: Kishimoto et al. with 8 RCTs

* As there was no other RCTmeta available, we decided to use Samara et al. study. However, we know that this study was about treatment-resistant schizophrenia.

# Appendix 5: Data sources of the current meta-analysis in the original publications (RWS)

**eTable 2: Data sources of the current meta-analysis in the original publications (RWS)**

| **List of RWS involved** | **Source of RR in the original paper** |
| --- | --- |
| Cooper et al. (2005)^12^ | Text^a^ |
| Ascher-Svanum et al. (2006)^13^ | Table 5 |
| Tiihonen et al. (2006)^4^ | Table 3 *(Adjusted Analysis)* |
| Haro et al. (2007)^14^ | Table 2 *(Discontinuation for any cause/Hazard ratio (95% CI))* |
| Kilzieh et al. (2008)^15^ | Text^b^ |
| Dossenbach et al. (2008)^16^ | Table 2 |
| Tiihonen et al. (2011)^17^ | Figure S1 *(Adjusted Hazard Ratio)* |
| Bitter et al. (2013)^18^ | Table 2 |
| Katona et al. (2014)^19^ | NA^c^ |
| Tiihonen et al. (2017)^20^ | Figure 3 |
| Takacs et al. (2019)^21^ | Table 4 *(Adjusted hazard ratio)* |

^a^ Text copied from the original paper^12^: *“Figure 1 shows survival curves of treatment discontinuation adjusted for age, sex, region, schizophrenia subtype, comorbidity, prior mental illness hospitalization, beneficiary type, substance use disorder, and prescriber specialty. We observed a high discontinuation rate in the first 30 days of treatment, indicating that many patients had filled one pre-scription only. The adjusted risk of discontinuing the index drug was lower for olanzapine users, compared with risperidone users (HR = 0.79; 95%CI, 0.74 to 0.84).”*^b^ Text copied from the original paper^15^: *“Discontinuation rates of index medication trials were high (73%) and lower for olanzapine (70%) than risperidone (76%) (P= 0.12). This trend for higher discontinuation rate of risperidone was observed in Cox regression analysis (hazards ratio = 1.23; 95% CI, 0.99–1.5).”*^c^ As no direct head-to-head comparisons of monotherapies were published in the paper of Katona *et. al.* (2014)^19^, for the purpose of current investigation we used the relevant unpublished results available from the original analysis.

# Appendix 6: Detailed results of current meta-analysis (RWS)

Here we present the results of our meta-analysis according to the strength of the evidence, i.e., based on the number of real-world studies that were available for a given pairwise comparison. Please note that due to the alphabetical listing of AP comparisons, RR values below 1 indicate risk reduction, whereas RR values above 1 indicate risk increase. Our summary presentation of the results is organized according to whether or not the evidence was statistically conclusive, and the individual studies pointed consistently in the same direction.

## Part 1: Comparisons with 3 or more RWS included (17 out of 25 comparisons)

1.2.2.1 Pairwise comparisons with statistically conclusive and consistent individual study outcomes

OLA-RIS: A total of 8 RWS were included in the meta-analysis. Among them, 6 showed a statistically significant superiority of OLA over RIS, while the remaining 2 studies^15,20^ yielded a numerical advantage for OLA. Our overall estimate for the decrease of relative risk of all-cause discontinuation with OLA as compared to RIS was significant with a value of RR=0.71 (95% CI=0.59-0.85).

OLA-QUE: A total of 5 comparisons from RWS were available. Of these, 4 favored OLA over QUE, while one study^20^ with no significant difference showed a numerical advantage for OLA. The overall estimate for the relative risk of risk reduction for all-cause discontinuation with OLA as compared to QUE was significant with an RR value of 0.72 (95% CI=0.56-0.92).

HAL-RIS: A total of 5 RWS were included in our meta-analysis. Among of them, 4 favored statistically significantly RIS over HAL; 1 study^17^ showed a numerical advantage for RIS. Our pooled result from the meta-analysis showed a statistically significant risk increase for HAL over RIS, with an RR value of 1.60 (95% CI=1.43-1.79).

RIS LAI-RIS: A total of 4 comparisons from RWS were available. All of them favored RIS LAI over RIS. The overall estimate for the relative risk of risk reduction for all-cause discontinuation with RIS LAI as compared to RIS was significant with an RR value of 0.60 (95% CI=0.50-0.73).

AMI-OLA: A total of 3 RWS were included in the meta-analysis. Each of them favored OLA statistically significantly. Our pooled result from the meta-analysis showed a statistically significant risk increase for AMI over OLA, with an RR value of 1.45 (95% CI=1.34-1.58).

ARI-OLA: A total of 3 comparisons from RWS were available. Among them, 2 favored statistically significantly OLA over ARI; 1 RWS^19^ showed a numerical advantage for OLA. Our pooled result from the meta-analysis revealed a statistically significant risk increase for ARI over OLA, with an RR value of 1.14 (95% CI=1.07-1.20).

ARI-RIS LAI: A total of 3 RWS were included in our meta-analysis. Of these, 2 of them favored RIS LAI statistically significantly over ARI; 1 study^19^ showed numerical advantage for RIS LAI. Our pooled result from the meta-analysis showed a statistically significant risk increase for ARI over RIS LAI, with an RR value of 1.26 (95% CI=1.13-1.42).

CLO-HAL: A total of 3 comparisons from RWS were available. All of them favored statistically significantly CLO over HAL. The overall estimate for the relative risk reduction for all-cause discontinuation with CLO as compared to HAL was significant with an RR value of 0.33 (95% CI=0.14-0.75).

AMI-RIS LAI: A total of 3 RWS were included in the meta-analysis. Each of them favored statistically significantly RIS LAI over AMI. Our pooled result from the meta-analysis showed a statistically significant risk increase for AMI over RIS LAI, with an RR value of 1.52 (95% CI=1.26-1.82).

CLO-RIS LAI: A total of 3 comparisons from RWS were available. All of them favored statistically significantly RIS LAI over CLO. The overall estimate for the relative risk increase for all-cause discontinuation with CLO as compared to RIS LAI was significant with an RR value of 1.36 (CI=1.12-1.65).

HAL-QUE: A total of 3 RWS were included in our meta-analysis. Of these, 2 of them favored statistically significantly QUE over HAL, 1 study^13^ showed numerical advantage for QUE. Our pooled result from the meta-analysis showed a statistically significant risk increase for HAL over QUE, with an RR value of 1.55 (95% CI=1.35-1.78).

QUE-RIS LAI: A total of 3 comparisons from RWS were available. Each of them favored statistically significantly RIS LAI over QUE. Our pooled result from the meta-analysis showed a statistically significant risk increase for QUE over RIS LAI with an RR value of 1.33 (95% CI=1.09-1.63).

1.2.2.2 Pairwise comparisons with statistically conclusive but with inconsistent individual study outcomes

HAL-OLA: A total of 5 RWS were available for the comparison. Of these, 4 studies favored statistically significantly OLA over HAL. One study^20^, however, found no difference between the two APs (RR=0.96 (95% CI=0.86-1.06)). Our pooled meta-analytic estimate from the five studies for the RR was 1.73 (CI=1.22-2.46).

1.2.2.3 Pairwise comparisons with neither statistically conclusive nor consistent individual outcomes

CLO-OLA: A total of 4 RWS were available for the comparison. Of these, 2 studies favored statistically significantly OLA over CLO. By contrast, 1 study^20^ indicated a highly statistically significant superiority of CLO over OLA. Furthermore, 1 study^14^ showed a numerical advantage of CLO. Due to the wide variation of individual study results, the pooled result of our meta-analysis was inconclusive, yielding no statistically significant or potentially clinically important difference between the two APs with an RR value of 0.96 (95% CI=0.63-1.47).

QUE-RIS: A total of 4 comparisons were included in our meta-analysis. Among them, 2 studies showed a statistically significant superiority of QUE over RIS; 2 studies^18,19^ found no difference between the two APs. Due to the highly variable individual study results, the pooled result of our meta-analysis was inconclusive, yielding no statistically significant difference but showed a numerical advantage of QUE with an RR value of 0.84 (95% CI=0.63-1.13).

OLA-RIS LAI: A total of 4 RWS were included in the meta-analysis. Of these, 3 of them favored statistically significantly RIS LAI over OLA; 1 study^21^ found numerical advantage of OLA. Due to the wide variation of individual study results, the pooled result of our meta-analysis was inconclusive, yielding no statistically significant difference but showed a very strong numerical advantage of RIS LAI with an RR value of RR=1.13 (95% CI=0.99-1.30).

CLO-RIS: A total of 3 comparisons from RWS were available. Among them, 2 studies showed a statistically significant superiority of CLO over RIS; by contrast, 1 study^19^ found statistically significant advantage of RIS over CLO. Due to the highly variable individual study results, the pooled result of our meta-analysis was inconclusive, yielding no statistically significant difference but showed a modest numerical advantage of CLO with an RR value of 0.76 (95% CI=0.42-1.38).

Those comparisons which have only 2 RWS included (8 out of 25 comparisons) we publish results in part 2 of this Appendix.

## Part 2: Comparisons with only 2 RWS included (8 out of 25 comparisons)

Conclusive and consistent

AMI-ARI: Both comparisons from RWS favored statistically significantly ARI over AMI. The overall estimate for the relative risk of risk increase for all-cause discontinuation with AMI as compared to ARI was significant with an RR value of 1.28 (CI=1.06-1.55).

AMI-QUE: Both comparisons from RWS favored statistically significantly QUE over AMI. The overall estimate for the relative risk increase for all-cause discontinuation with AMI as compared to QUE was significant with an RR value of 1.13 (CI=1.05-1.21).

ARI-RIS: Both comparisons from RWS favored statistically significantly RIS over ARI. The overall estimate for the relative risk of risk increase for all-cause discontinuation with ARI as compared to RIS was significant with an RR value of 1.27 (CI=1.13-1.42).

Inconclusive and consistent

AMI-RIS: Both comparisons from RWS found no statistically significant difference between the two APs. Among them, 1 study^18^ showed a very modest numerical advantage for AMI, while the other study^19^ found a very modest numerical advantage for RIS. Due to the different direction of the individual study results, the pooled result of our meta-analysis was inconclusive, yielding no statistically significant difference between the two APs with an RR value of 0.99 (95% CI=0.89-1.09).

ARI-QUE: None of the comparisons from RWS yielded a statistically significant difference between the two AP, even though both of them showed numerical advantage for ARI over QUE. The overall estimate for the relative risk reduction for all-cause discontinuation with ARI as compared to QUE was not statistically significant but a very strong numerical advantage with an RR value of 0.93 (CI=0.86-1.01).

Inconclusive and inconsistent

CLO-QUE: One study^18^ found no statistically significant difference between the two APs, while the other study^19^ showed statistically significant difference favoring QUE over CLO. Due to the different individual study results, the pooled result of our meta-analysis was inconclusive, yielding a numerical but statistically not significant difference between the two APs, favoring QUE over CLO with an RR value of 1.15 (95% CI=0.91-1.45).

AMI-CLO: One study^18^ found no statistically significant numerical difference between the two APs, while the other study^19^ showed statistically significant difference favoring AMI over CLO. Due to the different individual study results, the pooled result of our meta-analysis was inconclusive, yielding no statistically significant difference but numerical between the two APs favoring AMI over CLO with an RR value of 0.90 (95% CI=0.63-1.29).

ARI-CLO: One study^18^ found no statistically significant difference between the two APs, while the other study^19^ showed statistically significant difference favoring ARI over CLO. Due to the different study results, the pooled result of our meta-analysis was inconclusive, yielding a numerical, but statistically not significant difference between the two APs favoring ARI over CLO with an RR value of 0.83 (95% CI=0.57-1.19).

# Appendix 7: Basic descriptive statistics of previously published meta-analyses (RCTmeta)

**eTable 3: Basic descriptive statistics of previously published meta-analyses**

| **Authort** | **Title** | **Journal, and date of publication** | **Statistical Measure Calculated** | **AP pairs compared** | **Number of Trials^a^** | **Total Number of Patients^b^** | **Relative Risk** | **LCL of Relative Risk** | **UCL of Relative Risk** |
| --- | --- | --- | --- | --- | --- | --- | --- | --- | --- |
| Beasley CM Jr, Stauffer VL, Liu-Seifert H, Taylor CC, Dunayevich E, Davis JM.^5^ | All-cause treatment discontinuation in schizophrenia during treatment with olanzapine relative to other antipsychotics: an integrated analysis | J Clin Psychopharmacol. 2007 | Hazard Ratio | CLO-OLA | 3 | 409 | 1,20 | 0,90 | 1,60 |
|  |  |  |  | HAL-OLA | 5 | 948 | 1,40 | 1,20 | 1,70 |
|  |  |  |  | OLA-RIS | 5 | 421 | 0,77 | 0,63 | 0,91 |
| Soares-Weiser K, Béchard-Evans L, Lawson AH, Davis J, Ascher-Svanum H.^11^ | Time to all-cause treatment discontinuation of olanzapine compared to other antipsychotics in the treatment of schizophrenia: a systematic review and meta-analysis | Eur Neuropsychopharmacol. 2013 | Hazard Ratio | AMI-OLA | 3 | 1119 (791) | 1,15 | 0,93 | 1,43 |
|  |  |  |  | ARI-OLA | 2 | 1269 (566) | 1,23 | 1,08 | 1,41 |
|  |  |  |  | CLO-OLA | 4 | 596 (477) | 1,05 | 0,75 | 1,47 |
|  |  |  |  | HAL-OLA | 5 | 1651 (1112) | 1,54 | 0,93 | 2,56 |
|  |  |  |  | OLA-QUE | 6 | 3130 (1749) | 0,68 | 0,56 | 0,83 |
|  |  |  |  | OLA-RIS | 11 | 3482 (2117) | 0,80 | 0,71 | 0,90 |
| Samara MT, Dold M, Gianatsi M, Nikolakopoulou A, Helfer B, Salanti G, Leucht S.^9^ | Efficacy, Acceptability, and Tolerability of Antipsychotics in Treatment-Resistant Schizophrenia: A Network Meta-analysis | JAMA Psychiatry. 2016 | Odds Ratio | CLO-HAL | 3 | 646 | 0,53 | 0,29 | 1,12 |
|  |  |  |  | CLO-OLA | 7 | 956 | 1,28 | 0,76 | 2,22 |
|  |  |  |  | CLO-RIS | 6 | 587 | 0,97 | 0,54 | 1,72 |
|  |  |  |  | HAL-OLA | 5 | 731 | 1,92 | 1,03 | 4,17 |
|  |  |  |  | HAL-RIS | 2 | 145 | 0,87 | 0,31 | 2,44 |
|  |  |  |  | OLA-RIS | 2 | 112 | 0,66 | 0,22 | 2,03 |
| Sampson S, Hosalli P, Furtado VA, Davis JM^10^ | Risperidone (depot) for schizophrenia (Review) | Cochrane Database Syst Rev 2016 | Risk Ratio | ARI-RIS LAI | 2 | 723 | 1,20 | 0,77 | 1,89 |
|  |  |  |  | RIS LAI-RIS | 2 | 690 | 1,28 | 0,92 | 1,79 |
| Ostuzzi G, Bighelli I, So R, Furukawa TA, Barbui C.^8^ | Does formulation matter? A systematic review and meta-analysis of oral versus long-acting antipsychotic studies | Schizophr Res. 2017 | Risk Ratio | RIS LAI-RIS | 6 | 1151 | 1,17 | 0,95 | 1,44 |
| Krause M, Huhn M, Schneider-Thoma J, Rothe P, Smith RC, Leucht S.^7^ | Antipsychotic drugs for elderly patients with schizophrenia: A systematic review and meta-analysis | Eur Neuropsychopharmacol. 2018 | Odds Ratio | OLA-RIS | 3 | 281 | 0,54 | 0,31 | 0,93 |
| Kishimoto T, Hagi K, Nitta M, Kane JM, Correll CU.^6^ | Long-term effectiveness of oral second-generation antipsychotics in patients with schizophrenia and related disorders: a systematic review and meta-analysis of direct head-to-head comparisons | World Psychiatry. 2019 | Risk Ratio | AMI-OLA | 3 | 796 | 1,07 | 0,91 | 1,27 |
|  |  |  |  | ARI-OLA | 8 | 2117 | 1,17 | 1,05 | 1,30 |
|  |  |  |  | ARI-QUE | 2 | 522 | 0,75 | 0,38 | 1,45 |
|  |  |  |  | CLO-OLA | 4 | 1202 | 1,01 | 0,86 | 1,18 |
|  |  |  |  | CLO-RIS | 4 | 216 | 0,74 | 0,57 | 0,95 |
|  |  |  |  | OLA-QUE | 8 | 1942 | 0,79 | 0,71 | 0,89 |
|  |  |  |  | OLA-RIS | 16 | 3131 | 0,88 | 0,83 | 0,93 |
|  |  |  |  | QUE-RIS | 8 | 3227 | 1,07 | 0,98 | 1,18 |

^a^ Number of randomized clinical trials included in the meta-analysis for a given comparison.
^b^ The number of patients in Soares-Weiser *et. al.* paper provides the total number of patients included in the RCTs, regardless of the target APs compared. The authors of the current investigation looked up the original papers on the RCTs included in the meta-analysis by Soares-Weiser *et. al.*, and summed up only those number of patients who were allocated to the specific target drugs compared. The originally published numbers by Soares-Weiser are displayed in the Table’s cells; the numbers in the brackets are calculated by the authors.

# Appendix 8: Comparison of RWS with meta-analytic benchmarks from RCTs (RCTmeta)

**eTable 4: Comparison of RWS with meta-analytic benchmarks from RCTs**

| **Results of Pooled Estimates of RWS** | **Congruent with RCTmetas** | **Not congruent with RCTmetas** | **Row Total** |
| --- | --- | --- | --- |
| Statistically conclusive | 7 | 2 | 9 |
| Statistically not conclusive | 2 | 1 | 3 |
| Column Total | 9 | 3 | 12 |

# Appendix 9: Discussion about comparisons with statistically conclusive but inconsistent results

HAL-OLA: There was only one RWS which showed no difference between the two APs^20^, while the other 4 showed clear superiority for OLA over HAL.

CLO-OLA: In general, the findings from the studies included in the meta-analysis markedly differ from the results of the Tiihonen (2017) analysis. Hungarian data point in different direction than the data from West European analyses.

QUE-RIS: The findings, in general, from the studies included in the meta-analysis markedly differ from the results of the Tiihonen (2011) analysis, as they indicate no pair-wise difference with a large effect size, while the Tiihonen (2011) study showed a highly pronounced superiority of QUE over RIS.

OLA-RIS LAI: All studies showed statistically significant superiority of RIS LAI vs OLA with the exception of one^21^ which indicated no difference.

CLO-RIS: All studies showed statistically significant difference between the two APs; however, only one of the studies^17^ indicated a difference with large effect size (RR=0.43 (95% CI=0.35-0.54)) between these two medications.

The findings that show inconsistency in the above comparisons might be attributable to various factors including the regionally specific use of these APs, cohort effects, or differences in study methodology and data analysis. Regionally specific prescribing practice may explain the relative lack of effectiveness of olanzapine vs. haloperidol and risperidone vs quetiapine in the Finish population. It may also account for clozapine’s relative lack of effectiveness in the Hungarian population where clozapine is typically applied in low doses^18,19^. Discrepant RR estimate within the same country may also be explained by cohort effects, for example, the introduction of new LAIs in the Hungarian market^21^.

# Appendix 10: Results from the meta-analysis of Soares-Weiser *et. al.* (2013)

Pair-wise comparisons of olanzapine with 6 other APs, respectively, from RCTs and observational studies. Hazard-ratio (estimates with 95% confidence limits) for all-cause discontinuation data were extracted from the meta-analysis of Soares-Weiser *et. al.* (2013)^11^.

**eTable 5: Results from the meta-analysis of Soares-Weiser *et. al.* (2013)**

| **Olanzapine vs.** | **RCTs** | **Obs. Studies** |
| --- | --- | --- |
| Amisulpride | 0.87 (0.70-1.07) | 0.69 (0.53-0.90)* |
| Aripiprazole | 0.81 (0.71-0.93)* | 0.96 (0.84-1.10) |
| Clozapine | 0.95 (0.68-1.33) | 1.37 (0.99-1.89) |
| Haloperidol | 0.65 (0.39-1.07) | 0.56 (0.45-0.70)* |
| Quetiapine | 0.68 (0.56-0.83)* | 0.79 (0.58-1.08) |
| Risperidone | 0.80 (0.71-0.90)* | 0.83 (0.75-0.92)* |

Note: all results <1 are favorable to olanzapine and results >1 are favorable to the comparator antipsychotic medication.

# Appendix 11: RWS vs RCTs with incongruent results

With respect to HAL-RIS, our meta-analytic result indicated a clear advantage of RIS over HAL, but the meta-analysis of RCTs by Samara yielded no significant difference. We think that Samara et al’s results were limited by the fact that they could find only two input datasets from the previously published RCTs for the analysis^22,23^. Both of these studies had a relatively small sample size (Volavka *et. al.*’s study had 41 patients randomized to HAL and 37 patients to RIS; in Wirshing et al’s study there were 33 patients randomized to HAL and 34 patients to RIS), which may have limited the statistical power in these studies and hence in the subsequent meta-analysis.

As to the RIS LAI-RIS comparison, our meta-analytic finding based on RWS showed RIS LAI’s superiority over RIS, while primary and secondary benchmarks selected indicated that there was numerical but not significant difference favoring oral formulation. These results are congruent with several studies which report that LAI formulations do not show an advantage in clinical trial settings where treatment compliance is generally achieved by study personnel.

The comparison QUE vs RIS showed a numerical incongruency between the results of RWS and RCTmetas, the former suggesting a slight numerical, but statistically not significant, advantage for QUE, whereas the RCTmeta showed the opposite result. The fact that the RWS analysis was inconsistent with the RCTmeta might be due to regionally specific prescribing practice in the Finish population (please see discussion of this issue in Appendix 8).

# References

1. Mohamed, S., Rosenheck, R., Harpaz-Rotem, I., Leslie, D. & Sernyak, M. J. Duration of pharmacotherapy with long-acting injectable risperidone in the treatment of schizophrenia. *Psychiatr. Q.* **80**, 241–249 (2009)

2. DerSimonian R. & Laird N. Meta-analysis in clinical trials. *Control. Clin. Trials* **7**, 177–188 (1986)

3. Houwelingen, H. C., Arends, L. R. & Stijnen T. Advanced methods in meta-analysis: multivariate approach and meta-regression. *Stat. Med.* **21**, 589–624 (2002)

4. Tiihonen, J. *et. al.* Effectiveness of antipsychotic treatments in a nationwide cohort of patients in community care after first hospitalisation due to schizophrenia and schizoaffective disorder: observational follow-up study. *BMJ* **333**, 224. (2006) https://doi.org/10.1136/bmj.38881.382755.2F

5. Beasley, C. M. *et. al.* All-cause treatment discontinuation in schizophrenia during treatment with olanzapine relative to other antipsychotics: an integrated analysis. *J. Clin. Psychopharmacol.* **27**, 252–258 (2007)

6. Kishimoto, T., Hagi, K., Nitta, M., Kane, J. M. & Correll, C. U. Long-term effectiveness of oral second-generation antipsychotics in patients with schizophrenia and related disorders: a systematic review and meta-analysis of direct head-to-head comparisons. *World psychiatry* **18**, 208–224 (2019)

7. Krause, M. *et. al.* Antipsychotic drugs for elderly patients with schizophrenia: A systematic review and meta-analysis. *Eur. Neuropsychopharmacol.* **28**, 1360–1370 (2018)

8. Ostuzzi, G., Bighelli, I., So, R., Furukawa, T. A. & Barbui, C. Does formulation matter? A systematic review and meta-analysis of oral versus long-acting antipsychotic studies. *Schizophr. Res.* **183**, 10–21 (2017)

9. Samara, M. T. *et. al.* Efficacy, Acceptability, and Tolerability of Antipsychotics in Treatment-Resistant Schizophrenia: A Network Meta-analysis. *JAMA Psychiatry* **73**, 199–210 (2016)

10. Sampson, S., Hosalli, P., Furtado, V. A. & Davis, J. M. Risperidone (depot) for schizophrenia. *Cochrane Database Syst. Rev.* **4**, CD004161 (2016)

11. Soares-Weiser, K., Béchard-Evans, L., Lawson, A. H., Davis, J. & Ascher-Svanum, H. Time to all-cause treatment discontinuation of olanzapine compared to other antipsychotics in the treatment of schizophrenia: a systematic review and meta-analysis. *Eur. Neuropsychopharmacol.* **23**, 118–125 (2013)

12. Cooper D., Moisan J., Gaudet M., Abdous B. & Grégoire J.P. Ambulatory use of olanzapine and risperidone: a population-based study on persistence and the use of concomitant therapy in the treatment of schizophrenia. *Can. J. Psychiatry* **50**, 901-8 (2005)

13. Ascher-Svanum H. *et. al.* Time to discontinuation of atypical versus typical antipsychotics in the naturalistic treatment of schizophrenia. *BMC Psychiatry* **6**, 8 (2006) https://doi.org/10.1186/1471-244X-6-8

14. Haro, J. M. *et. al.* Three-year antipsychotic effectiveness in the outpatient care of schizophrenia: observational versus randomized studies results. *Eur. Neuropsychopharmacol.* **17**, 235–244 (2007)

15. Kilzieh, N., Todd-Stenberg, J. A., Kennedy, A., Wood, A. E., & Tapp, A. M. Time to discontinuation and self-discontinuation of olanzapine and risperidone in patients with schizophrenia in a naturalistic outpatient setting. *J. Clin. Psychopharmacol.* **28**, 74–77 (2008)

16. Dossenbach, M. *et. al.* Long-term antipsychotic monotherapy for schizophrenia: disease burden and comparative outcomes for patients treated with olanzapine, quetiapine, risperidone, or haloperidol monotherapy in a pan-continental observational study. *J. Clin. Psychiatry* **69**, 1901–1915 (2008)

17. Tiihonen, J. *et al.* A nationwide cohort study of oral and depot antipsychotics after first hospitalization for schizophrenia. *Am. J. Psychiatry* **168**, 603–609 (2011)

18. Bitter I. *et. al.* Comparative effectiveness of depot and oral second generation antipsychotic drugs in schizophrenia: a nationwide study in Hungary. *Eur. Neuropsychopharmacol.* **23**, 1383–1390 (2013)

19. Katona, L., Czobor, P. & Bitter, I. Real-world effectiveness of antipsychotic monotherapy vs. polypharmacy in schizophrenia: to switch or to combine? A nationwide study in Hungary. *Schizophr. Res.* **152**, 246–254 (2014)

20. Tiihonen, J. *et. al.* Real-World Effectiveness of Antipsychotic Treatments in a Nationwide Cohort of 29 823 Patients With Schizophrenia. *JAMA Psychiatry* **74**, 686–693 (2017)

21. Takács, P. *et. al.* Comparative effectiveness of second generation long-acting injectable antipsychotics based on nationwide database research in Hungary. *PloS ONE* **14**, e0218071 (2019) https://doi.org/10.1371/journal.pone.0218071

22. Volavka, J. *et. al.* Clozapine, olanzapine, risperidone, and haloperidol in the treatment of patients with chronic schizophrenia and schizoaffective disorder. *Am. J. Psychiatry* **159**, 255–262 (2002)

23. Wirshing, D. A. *et. al.* Risperidone in treatment-refractory schizophrenia. *Am. J. Psychiatry* **156**, 1374–1379 (1999)
